# MRI-based deep learning radiomics to differentiate dual-phenotype hepatocellular carcinoma from HCC and intrahepatic cholangiocarcinoma: a multicenter study

**DOI:** 10.1186/s13244-025-01904-y

**Published:** 2025-01-29

**Authors:** Qian Wu, Tao Zhang, Fan Xu, Lixiu Cao, Wenhao Gu, Wenjing Zhu, Yanfen Fan, Ximing Wang, Chunhong Hu, Yixing Yu

**Affiliations:** 1 Department of Radiology, The First Affiliated Hospital of Soochow University, Suzhou, China; 2 Department of Radiology, Affiliated Nantong Hospital 3 of Nantong University, Nantong, China; 3 Cancer Center, Zhongshan Hospital, Fudan University, Shanghai, China; 4 Department of Nuclear Medical Imaging, Tangshan People’s Hospital, Tangshan, China; 5 The First People’s Hospital of Taicang, Taicang, China

**Keywords:** Liver cancer, Magnetic resonance imaging, Radiomics, Deep learning, Differential diagnosis

## Abstract

**Objectives:**

To develop and validate radiomics and deep learning models based on contrast-enhanced MRI (CE-MRI) for differentiating dual-phenotype hepatocellular carcinoma (DPHCC) from HCC and intrahepatic cholangiocarcinoma (ICC).

**Methods:**

Our study consisted of 381 patients from four centers with 138 HCCs, 122 DPHCCs, and 121 ICCs (244 for training and 62 for internal tests, centers 1 and 2; 75 for external tests, centers 3 and 4). Radiomics, deep transfer learning (DTL), and fusion models based on CE-MRI were established for differential diagnosis, respectively, and their diagnostic performances were compared using the confusion matrix and area under the receiver operating characteristic (ROC) curve (AUC).

**Results:**

The radiomics model demonstrated competent diagnostic performance, with a macro-AUC exceeding 0.9, and both accuracy and F1-score above 0.75 in the internal and external validation sets. Notably, the vgg19-combined model outperformed the radiomics and other DTL models. The fusion model based on vgg19 further improved diagnostic performance, achieving a macro-AUC of 0.990 (95% CI: 0.965–1.000), an accuracy of 0.935, and an F1-score of 0.937 in the internal test set. In the external test set, it similarly performed well, with a macro-AUC of 0.988 (95% CI: 0.964–1.000), accuracy of 0.875, and an F1-score of 0.885.

**Conclusions:**

Both the radiomics and the DTL models were able to differentiate DPHCC from HCC and ICC before surgery. The fusion models showed better diagnostic accuracy, which has important value in clinical application.

**Critical relevance statement:**

MRI-based deep learning radiomics were able to differentiate DPHCC from HCC and ICC preoperatively, aiding clinicians in the identification and targeted treatment of these malignant hepatic tumors.

**Key Points:**

- Fusion models may yield an incremental value over radiomics models in differential diagnosis.
- Radiomics and deep learning effectively differentiate the three types of malignant hepatic tumors.
- The fusion models may enhance clinical decision-making for malignant hepatic tumors.

**Graphical Abstract:**

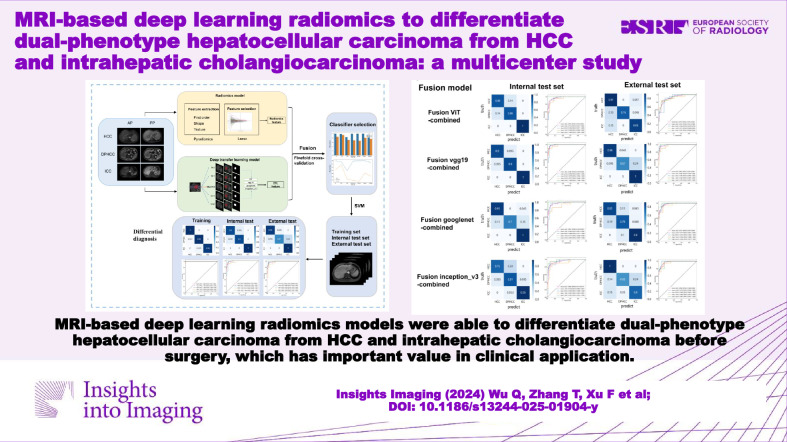

## Introduction

The World Health Organization (WHO) classifies primary liver cancer into three main entities: hepatocellular carcinoma (HCC), intrahepatic cholangiocarcinoma (ICC), and combined hepatocellular and cholangiocarcinoma (cHCC-CC). HCC is the most common type, accounting for 75–85% of primary liver cancer and ranking third in the world in terms of cancer-related deaths. ICC is the second most common type of primary liver cancer, accounting for 10–15% of all cases [1]. The WHO defines cHCC-CC as the presence of both classical HCC and ICC components in a single nodule, with a transitional or intermediate region between the two tumor components [2].

Unlike cHCC-CC, dual-phenotype hepatocellular carcinoma (DPHCC), a subtype of HCC, has typical HCC morphological features, but 15% of its cells strongly co-express HCC markers (HepPar-1, Glypican-3) and ICC markers (CK19, CK7) [3]. Due to the dual biological behavior of HCC and ICC, DPHCC was first discussed in 2011. DPHCC is highly invasive, proliferative, and migratory, accounting for about 10% of all HCC cases [3]. CK19 is considered a marker of cancer stem cells (CSCs), and its positive expression is a typical characteristic of DPHCC [4]. The responses of CK19^+^ and CK19^−^ HCC to different chemotherapeutic drugs are quite different, and CK19^+^ HCC has been confirmed to be resistant to chemotherapy, such as 5-fluorouracil and doxorubicin [5–7]. The presence of tumors without a capsule and those with irregular margins is notably more common in patients with DPHCC compared to those with non-DPHCC. During surgical resection, DPHCC patients often necessitate a wider surgical margin to ensure adequate clearance, while the efficacy of radiofrequency ablation (RFA) may also be compromised in DPHCC patients with incomplete capsules compared to those with complete capsules [8, 9]. In addition, studies have shown that CK19-positive HCC is associated with a higher recurrence rate following radiofrequency ablation [10]. According to the 2019 WHO classification of tumors, CK19-positive HCC displays significantly greater resistance to conventional transarterial chemoembolization (cTACE), with evidence suggesting that CK19-positive HCC patients exhibit poorer treatment responses to cTACE [11]. Hence, preoperative diagnosis and personalized therapy are essential. However, in the absence of histopathology and immunohistochemistry, clinical differential diagnosis of these three entities is extremely difficult, and preoperative misdiagnosis can mislead treatment decisions. In recent years, researchers have attempted to diagnose DPHCC by combining MRI imaging features with clinical laboratory features [9, 12].

Radiomics is based on quantitative image description, which quantifies a tumor’s morphological appearance, or imaging phenotype, using mathematical quantitative features [13, 14]. Radiologists find it challenging to assess this kind of quantitative data, but sophisticated computer programs can calculate it. It has been frequently utilized as a technique to evaluate tumor heterogeneity since it was made famous by Lambin et al in 2012 in order to establish a correlation with clinical or biological data [15–17].

Deep learning (DL) is a branch of machine learning, including transformer and convolutional neural networks (CNN). Vision transformer (ViT), a type of transformer, shows state-of-the-art performance in many medical studies [18, 19]. CNN is also a widely used deep learning method in image processing applications [20, 21]. CNN is a powerful nonlinear approach that can automatically learn and extract the hierarchical structural features of data [22, 23]. Vgg19 is a deep neural network with 19 layers. It utilizes small 3 × 3 convolutional filters uniformly. GoogLeNet introduced inception modules featuring multiple parallel paths to capture information at different scales. This design aims to prevent overfitting and enhance computational efficiency. Inception_v3 represents an evolution of GoogLeNet, refining the inception module and introducing factorized convolutions. It strikes a balance between model complexity and computational efficiency. Deep learning technology is increasingly being used in medical imaging to solve various segmentation or classification tasks [24, 25]. Jiang et al reported the ViT model based on MRI could predict survival in patients with rectal cancer [26]. Oestmann PM et al reported the CNN deep learning approach capable of differentiating between HCC and non-HCC lesions on multi-phasic contrast-enhanced MRI (CE-MRI) [27]. However, there is a lack of an automated machine learning-based diagnostic model for preoperative discrimination among DPHCC, HCC, and ICC.

Acquiring a significant number of medical images presents a challenge. Therefore, leveraging a pre-trained model such as deep transfer learning (DTL) can enhance the model’s generalization ability, especially when confronted with a limited training dataset. In recent years, DTL has been gradually applied to various medical image analysis fields [28–30]. In this study, we developed and validated the DTL models to differentiate DPHCC from HCC and ICC based on ViT, vgg19, googlenet, and inception_v3 architectures. We compared the diagnosis efficacy of DTL models with radiomics models. Additionally, we used the DTL and radiomics features to establish the fusion models and evaluated their predictive performance.

## Materials and methods

### Patients

**Fig. 1:**
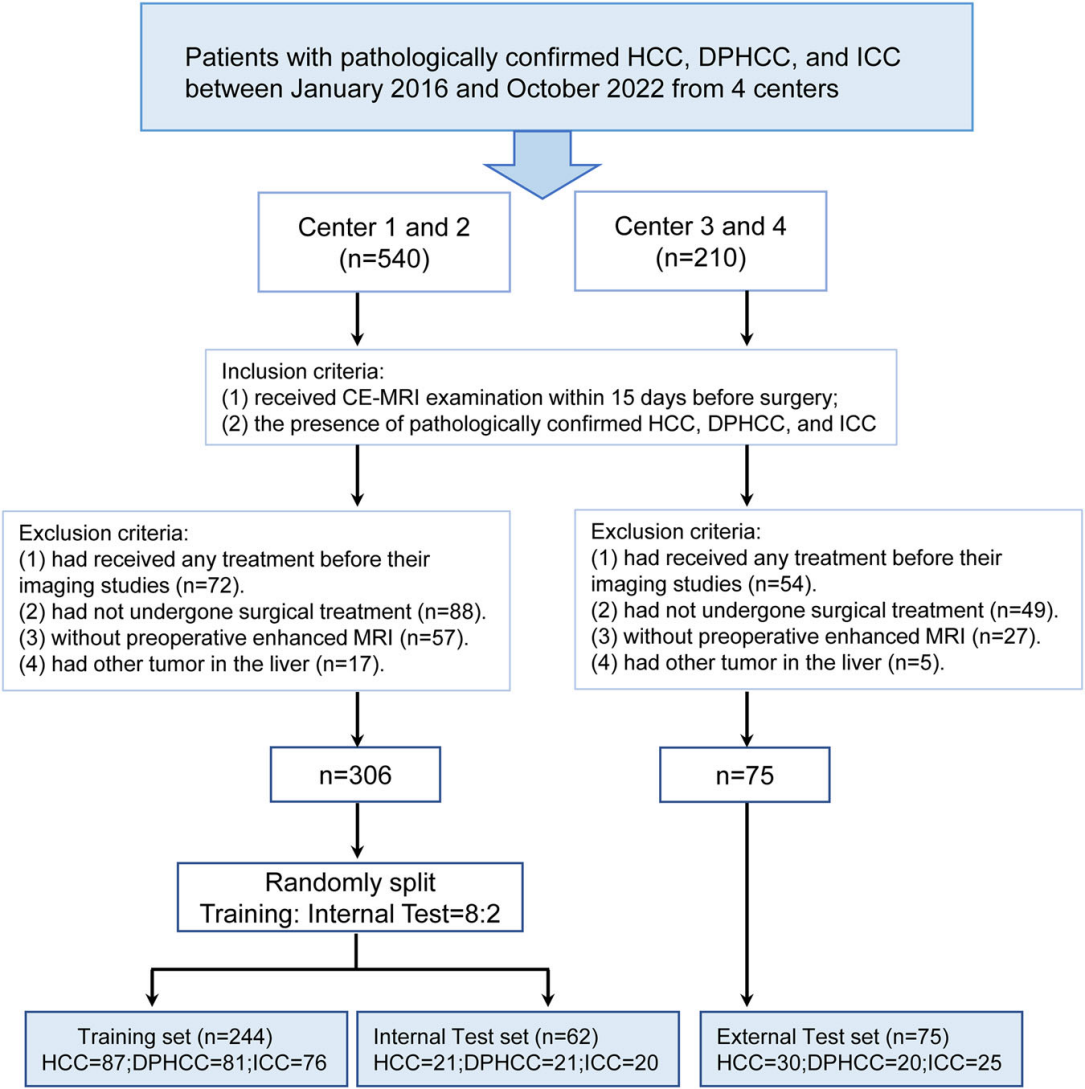
Patients’ flowchart for this study. Key inclusion/exclusion criteria for the study. HCC, hepatocellular carcinoma; DPHCC, dual-phenotype hepatocellular carcinoma; ICC, intrahepatic cholangiocarcinoma; CE-MRI, contrast-enhanced MRI

The Institutional Ethics Review Board approved the retrospective study (ethics approval number: 2021331) and waived the requirement for written informed consent. This study consecutively included patients from the First Affiliated Hospital of Soochow University (center 1), the Affiliated Nantong Hospital 3 of Nantong University (center 2), the Tangshan People’s Hospital (center 3), and the First People’s Hospital of Taicang (center 4) who received CE-MRI from January 2016 to October 2022. Inclusion criteria: (1) received CE-MRI examination within 15 days before surgery; (2) the presence of pathologically confirmed DPHCC, HCC, and ICC. Exclusion criteria: (1) received preoperative antitumor therapy; (2) had not undergone surgical treatment; (3) incomplete clinical or pathological information; and (4) had another tumor in the liver. A total of 381 patients (267 males and 114 females, mean age 61 years, range of 30–85 years) were included in this study, including 138 patients with HCC, 122 patients with DPHCC, and 121 patients with ICC. Clinical data and preoperative routine laboratory results were collected, including age, sex, viral hepatitis status, cirrhosis, alpha-fetoprotein (AFP), aspartate aminotransferase (AST), alanine aminotransferase (ALT), γ-glutamyl transferase (GGT), Child-Pugh class, maximum tumor diameter and number of tumors. The patients from centers 1 and 2 were divided into a training set and internal testing set at a ratio of 8:2 via stratified random sampling, and the patients from centers 3 and 4 were used as an independent external testing set. A flowchart of patient inclusion and exclusion was shown in Fig.1 in this study.

### Histopathology and immunohistochemistry

All of the following criteria needed to be fulfilled for the pathological diagnosis of DPHCC. (1) HCC was histologically diagnosed according to morphologic criteria defined by WHO. (2) Immunohistochemical results showed that at least one hepatocyte marker, such as HepPar-1 or Glypican-3, was strongly positive in more than 15% of tumor cells, with mainly diffuse distribution. (3) At least one bile duct cell marker, such as CK19 or CK7, was strongly positive in more than 15% of tumor cells. The pathological assessment was independently and qualitatively performed by two pathologists (M.Z.D., reader 1, with more than 10 years of experience in pathology, and L.C.G., reader 2, with more than 15 years of experience in pathology).

### CE-MRI images acquisition

CE-MRI images from four centers were acquired by using Simens Magnetom Skyra 3.0-T MRI (Siemens, Germany), GE Medical Systems 3.0-T MRI (GE, USA), Philips Medical Systems 3.0-T MRI (Philips Netherlands) imaging system, and 16-channel abdominal coils. The detailed sequences and parameters of the CE-MRI scan are shown in Supplementary Material 1.

### Radiomics model development

The arterial phase (AP) and portal venous phase (PP) CE-MRI images of 381 patients, acquired from different machines with varying imaging protocols, were normalized to 0-255 and subjected to histogram equalization. Then the images were imported into the publicly available ITK-SNAP software (version 4.0.0, www.itksnap.org). Two radiologists (Q.W. and Y.Y.), with 5 years and 10 years of professional experience, respectively, manually segmented the volumes of interest (VOIs) under the supervision of a board-certified subspecialist (C.H.), who has 20 years of professional experience in liver cancer.

The “PyRadiomics” python package (http://www.radiomics.io/pyradiomics.html) was used to extract the radiomics features from the VOIs. Radiomic features were derived from both the original and pre-processed images using wavelet and Laplacian of Gaussian (LoG) filters (sigma = 2.0, 3.0, 4.0, and 5.0). Feature extraction was carried out on images that were resampled to voxel dimensions of 1 × 1 × 1 mm³, with an intensity bin width of 5 for discretization. The 1781 radiomics features were extracted from the VOIs of AP and PP, respectively, including first-order statistics, shape, and texture features. Texture features were computed using the gray level co-occurrence matrix, gray level run length matrix, gray level size zone matrix, gray level dependence matrix, and neighboring gray-tone difference matrix. After *Z*-score normalization, the least absolute shrinkage and selection operator (LASSO) was applied to reduce the dimension of the features and perform the feature selection (Fig. S1). Five-fold cross-validation was used to select the value of the penalization parameter lambda (λ) that resulted in the sparsest model while remaining within one standard error of the minimum loss. Finally, we developed the AP, PP, and combined (AP + PP) radiomics models and compared the performance of different classifiers, including the support vector machine (SVM), logistic regression (LR), random forest (RF), and decision tree (DT), in differentiating DPHCC from HCC and ICC. The differential diagnosis efficiency of radiomics models was assessed by the area under the receiver operating characteristic (ROC) curve (AUC) and confusion matrices.

### Deep learning model development

Comprehensive 2D signal intensity information was extracted from the CE-MRI images of 381 patients. The 2D MAX-ROI plane was determined by calculating the area of non-zero pixels in each layer of the 3D VOI, along with the inclusion of adjacent upper and lower four MRI slices, ensuring comprehensive signal intensity extraction from the tumor. The axial slice with the most tumor area was automatically selected as the “maximum region of interest (Max-ROI)” slice, and the other four images were extracted from the Max-ROI images from 2 upper (+2), 1 upper (+1), 1 lower (−1) and 2 lower (−2). We carefully confirmed the selected adjacent upper and lower slices all containing tumor tissue. The tumor’s region of five images was precisely cropped to the minimum bounding rectangle image, and each instance was saved as a distinct image for subsequent feature extraction. Five 2D images were extracted from the AP and PP CE-MRI images of each patient, respectively. Finally, the 3810 cropped 2D images were obtained of the 381 patients.

The ViT model and CNN models, including vgg19, googlenet, and inception_v3, were pretrained on the ImageNet dataset for DTL.DTL features were extracted from a penultimate layer in the pretrained DTL models. Finally, for each 2D image, 1024, 4096, 1024, and 2048 DTL features were extracted from the penultimate layer of the fine-tuned four models for ViT, vgg19, googlenet, and inception_v3, respectively. All the segmentation, pretraining, and DTL feature extraction were performed on a single NVIDIA Quadro RTX 3060Ti 8GB GPU in Windows 11. Similarly, *Z*-score and LASSO were performed to achieve feature normalization and selection. 12 DLT models (ViT-AP, ViT-PP, ViT-combined, vgg19-AP, vgg19-PP, vgg19-combined, googlenet-AP, googlenet-PP, googlenet-combined, inception_v3-AP, inception_v3-PP, and inception_v3-combined models) were built using the “PyTorch1.8.1” framework. The performance of various classifiers, including SVM, LR, RF, and DT, was compared using ROC curves and confusion matrices to identify the optimal classifier.

**Fig. 2:**
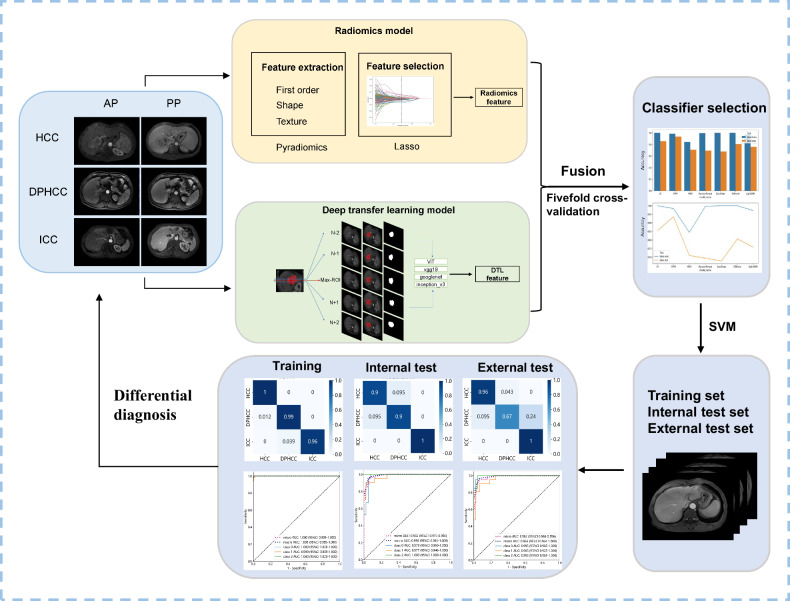
Radiomics and DTL models pipelines. The radiomics and DTL systems were developed separately and fusion the radiomics and DTL features to construct the fusion models. The performances were compared based on the ROC curve and confusion matrix of training, internal test, and external test sets. HCC, hepatocellular carcinoma; DPHCC, dual-phenotype hepatocellular carcinoma; ICC, intrahepatic cholangiocarcinoma; DTL, deep transfer learning; ViT, vision transformer; AP, arterial phase; PP, portal venous phase; LASSO, least absolute shrinkage and selection operator; SVM, support vector machine; ROC, receiver operating characteristic; AUC, area under the curve

We fused radiomics and deep learning features to develop 12 fusion models: Fusion-ViT-AP, Fusion-ViT-PP, Fusion-ViT-combined, Fusion-vgg19-AP, Fusion-vgg19-PP, Fusion-vgg19-combined, Fusion-googlenet-AP, Fusion-googlenet-PP, Fusion-googlenet-combined, Fusion-inception_v3-AP, Fusion-inception_v3-PP, and Fusion-inception_v3-combined models. The different classifiers selection and assessment of differential diagnosis efficiency based on the ROC curves and confusion matrices. The model workflow is shown in Fig. 2.

### Statistical analysis

Statistical analyses were performed using R software (version 4.1.0, https://www.r-project.org/). To evaluate the interobserver agreement for feature extraction, inter-class correlation coefficient analysis was performed on 30 randomly selected cases that were delineated by two radiologists. After two weeks, one of the radiologists repeated the segmentation of 30 cases to assess the intra-class correlation coefficient (ICC). Inter-class correlation coefficients or ICCs greater than 0.8 were considered as high stability features. Consistency levels were categorized as low (ICCs < 0.5), moderate (ICCs between 0.5 and < 0.8), and high (ICCs ≥ 0.8). The remaining cases were delineated by the more experienced radiologist. The LASSO regression, SVM, LR, RF, and DT classifier, confusion matrix, and ROC curve analysis were performed by the “sklearn” Python package. The DTL models were implemented using the “PyTorch 1.8.1” deep learning framework. The prediction performance was evaluated using accuracy, micro-AUC, macro-AUC, precision, recall, F1-score, and the confusion matrix. The categorical variables were compared by chi-square tests, and the comparison between quantitative variables was performed using one-way ANOVA or Kruskal-Wallis’s test. The differential diagnosis of three types of malignant hepatic tumors examples was visualized by the Gradient-weighted Class Activation Mapping (Grad-CAM) heatmaps using Python. Additionally, the correlation was evaluated using the Spearman rank correlation test. *p* < 0.05 indicated a significant difference.

## Results

### Baseline characteristics

**Fig. 3:**
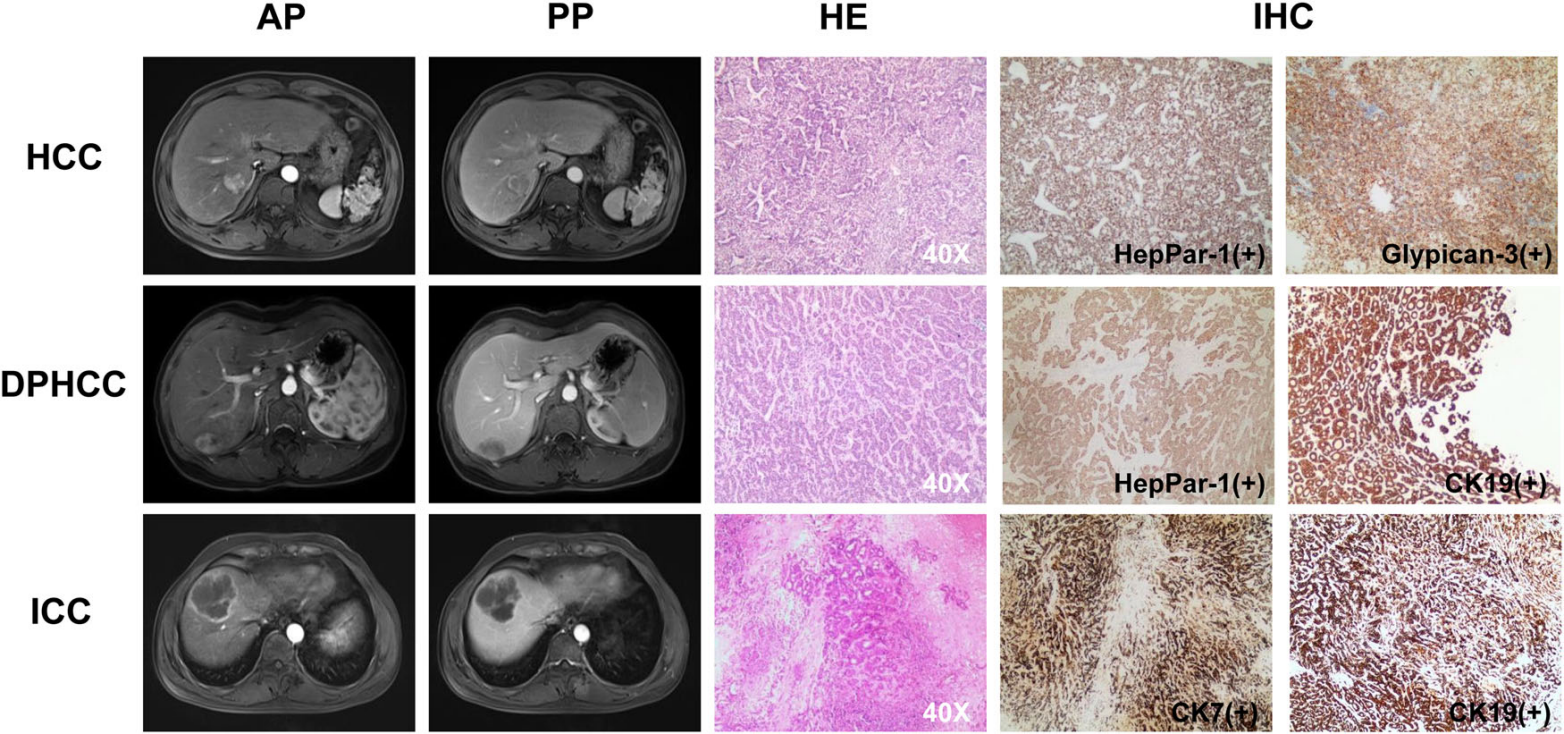
HCC, DPHCC, and ICC examples. Imaging examples comprise AP and PP MRI, accompanied by corresponding HE and IHC staining for HCC, DPHCC, and ICC. HE staining elucidated the hepatocytic origin of both HCC and DPHCC, whereas ICC originated from cholangiocytes. IHC staining demonstrated the presence of HepPar-1(+) and Glypican-3(+) in HCC, Glypican-3(+) and CK19(+) in DPHCC, and CK7(+) and CK19(+) in ICC. HCC, hepatocellular carcinoma; DPHCC, dual-phenotype hepatocellular carcinoma; ICC, intrahepatic cholangiocarcinoma; AP, arterial phase; PP, portal venous phase; HE, hematoxylin-eosin; IHC, immunohistochemistry

According to the ratio of 8:2, a total of 306 patients (centers 1 and 2) were randomly divided into a training set (*n* = 244, 81 DPHCC; 87 HCC; 76 ICC)(Fig. 3), including 179 males and 65 females, median age 60 years, and an internal test set (*n* = 62, 21 DPHCC; 21 HCC; 20 ICC), including 43 males, and 19 females, median age 62 years. The patients from centers 3 and 4 were used as an external test set (*n* = 75, 30 HCC, 20 DPHCC, and 25 ICC), including 45 males and 30 females, median age of 63 years.

The patient’s recruitment workflow is shown in Fig. 1. In the training set, there were significant differences in age, sex, AST, AFP, HBV, cirrhosis, and diameter (all *p* < 0.05). In the internal test set, age, sex, AFP, GGT, HBV, and cirrhosis showed significant differences (all *p* < 0.05). In the external test set, age, sex, AFP, GGT, HBV, cirrhosis, and diameter showed significant differences (all *p* < 0.05). Detailed clinical characteristics of the 381 patients are presented in Table 1.

**Table 1:** Baseline characteristics in the training, internal test, and external test sets

Characteristics	Training set (*n* = 244)			*p* value	Internal test set (*n* = 62)			*p* value	External test set (*n* = 75)			*p* value
	HCC (*n* = 87)	DPHCC (*n* = 81)	ICC (*n* = 76)		HCC (*n* = 21)	DPHCC (*n* = 21)	ICC (*n* = 20)		HCC (*n* = 30)	DPHCC (*n* = 20)	ICC (*n* = 25)	
Age	58 (51, 66)	57 (52, 64)	64 (55, 69)	0.007	56.95 ± 14.02	58.86 ± 10.40	66.00 ± 8.87	0.034	60 (56, 68)	61 (49, 66)	68 (60, 73)	0.038
Sex				< 0.001				0.007				0.042
Male	72	65	42		19	15	9		23	11	11	
Famale	15	16	34		2	6	11		7	9	14	
ALT (U/L)				0.116				0.927				0.147
≤ 50	63	63	65		17	17	17		25	15	15	
> 50	24	16	11		4	4	3		5	5	10	
AST (U/L)				0.014				0.889				0.452
≤ 40	56	48	61		16	17	15		21	11	14	
> 40	31	33	15		5	4	5		9	9	11	
AFP (ug/L)				< 0.001				< 0.001				< 0.001
≤ 25	38	44	64		8	9	19		8	8	23	
> 25	49	37	12		13	12	1		20	12	2	
GGT (U/L)				0.225				0.038				< 0.001
≤ 60	42	43	30		12	13	5		23	12	5	
> 60	45	38	46		9	8	15		7	8	20	
HBV				< 0.001				< 0.001				< 0.001
No	8	25	53		4	4	14		13	2	19	
Yes	79	56	23		17	17	6		17	18	6	
Cirrhosis				< 0.001				< 0.001				< 0.001
No	38	42	69		7	8	18		15	14	24	
Yes	49	39	7		14	13	2		15	6	1	
Diameter (mm)	58 (50, 66)	33 (20, 58)	63 (42, 87)	< 0.001	50.41 ± 18.85	53.19 ± 38.99	62.33 ± 28.72	0.421	42 (23, 63)	32 (19, 45)	64 (41, 88)	< 0.001
Multifocal				0.072				0.849				0.223
No	65	64	48		18	18	16		22	14	13	
Yes	22	17	28		3	3	4		8	6	12	
Child-Pugh class				0.824				0.889				0.974
A	63	62	56		16	17	15		22	15	19	
B	24	19	20		5	4	5		8	5	6	

*HCC* hepatocellular carcinoma, *DPHCC* dual-phenotype hepatocellular carcinoma, *ICC* intrahepatic cholangiocarcinoma, *AFP* alpha-fetoprotein, *ALT* alanine aminotransferase, *AST* aspartate aminotransferase, *GGT* gamma-glutamyl transferase, *HBV* hepatitis B virus

### Radiomics models construction

**Fig. 4:**
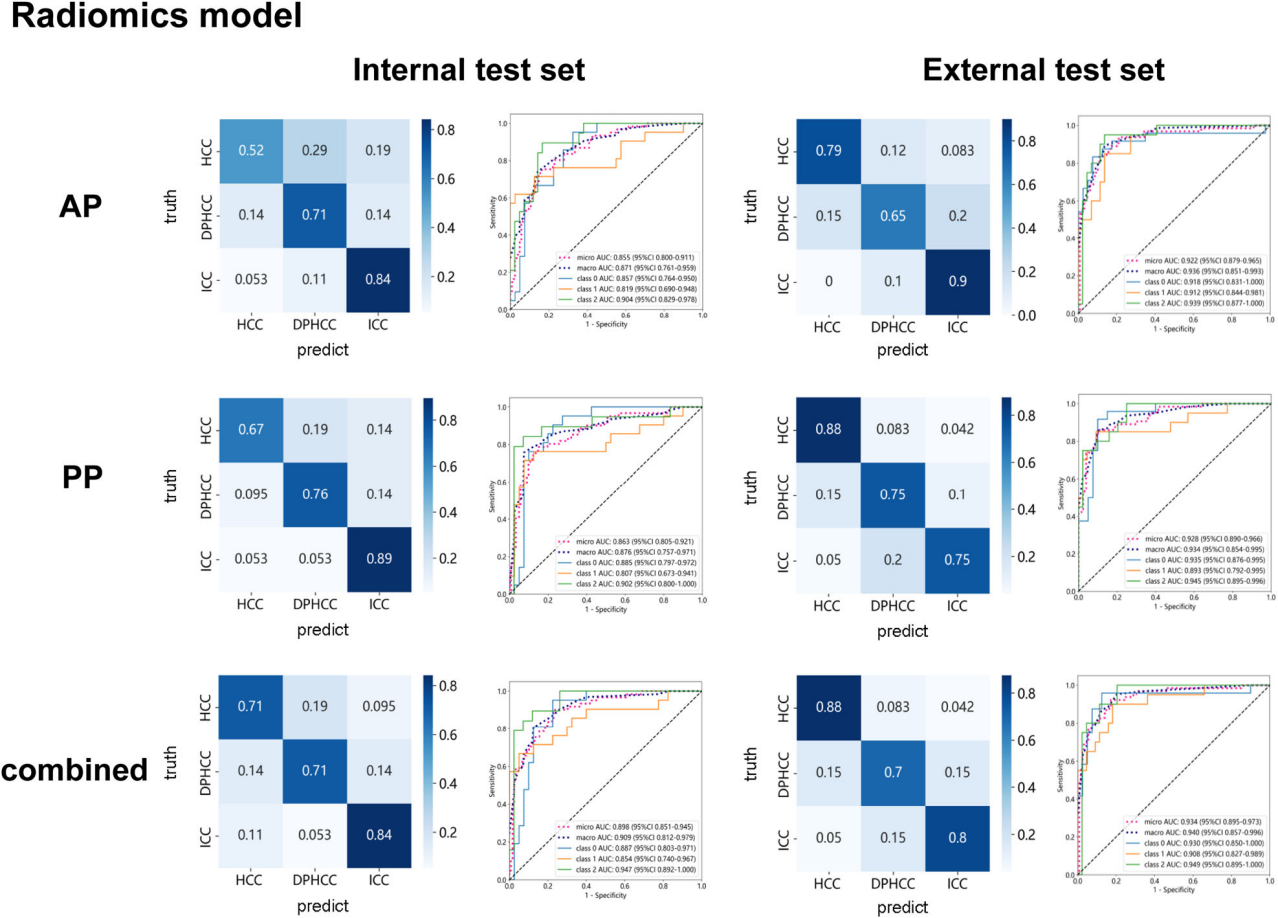
Differential diagnosis of HCC, DPHCC, and ICC based on radiomics models. Confusion matrices and ROC curves of AP, PP, and combined radiomics models in the internal and external test sets. Class 0, HCC; class 1, DPHCC; and class 2, ICC. HCC, hepatocellular carcinoma; DPHCC, dual-phenotype hepatocellular carcinoma; ICC, intrahepatic cholangiocarcinoma; AP, arterial phase; PP, portal venous phase; ROC, receiver operating characteristic; AUC, area under the curve. CI, confidence interval

The 1781 AP and PP radiomics features were extracted, respectively. The average inter-class correlation coefficient between the two radiologists for VOI delineation was 0.85, and the average ICC was 0.88, demonstrating satisfactory reproducibility in feature extraction. The radiomics feature selection and reduction are shown in Fig. S1. The results of the confusion matrices and ROC curves for differentiating DPHCC from HCC and ICC of internal and external test sets are shown in Fig. 4. The combined radiomics model has better prediction performance. The macro-AUC (95% CI), accuracy, and F1-score of the combined radiomics model were 0.976 (0.954–0.994), 0.878, and 0.881 in the training set, 0.909 (0.812–0.979), 0.754, and 0.759 in the internal test set, and 0.940 (0.857–0.996), 0.797, and 0.799 in the external test set (Table 2).

**Table 2:** Performance of radiomics models

Radiomics model		Macro-AUC (95% CI)	Accuracy	Precision	Recall	F1-score
AP						
	Training	0.949 (0.915–0.977)	0.845	0.850	0.845	0.847
	Internal test	0.871 (0.761–0.959)	0.689	0.698	0.696	0.697
	External test	0.936 (0.851–0.993)	0.781	0.774	0.780	0.777
PP						
	Training	0.936 (0.900–0.967)	0.804	0.799	0.799	0.799
	Internal test	0.876 (0.757–0971)	0.770	0.778	0.776	0.777
	External test	0.934 (0.854–0.995)	0.797	0.804	0.802	0.803
Combined						
	Training	0.976 (0.954–0.994)	0.878	0.884	0.878	0.881
	Internal test	0.909 (0.812–0.979)	0.754	0.758	0.760	0.759
	External test	0.940 (0.857–0.996)	0.797	0.800	0.799	0.799

*AP* arterial phase, *PP* portal venous phase, *Macro-AUC* macro-average area under the curve, CI confidence interval

### DTL model construction and selection

To find the most suitable model for the differential diagnosis of DPHCC from HCC and ICC, we developed the 12 types of DTL models using AP, PP, and combined DTL features, respectively, and compared their diagnostic performance. The vgg19-combined model showed better diagnostic accuracy. The macro-AUC (95% CI), accuracy, and F1-score of the vgg19-combined model were 0.999 (0.996–1.000), 0.960, and 0.958 in the training set, 0.982 (0.954–1.000), 0.920, and 0.921 in the internal test set, and 0.934 (0.863–0.986), 0.828, and 0.829 in the external test set (Figs. S2–S5 and Table 3).

**Table 3:** Performance of DTL models

DTL model		Macro-AUC (95% CI)	Accuracy	Precision	Recall	F1-score
ViT						
AP	Training	0.860 (0.807–0.907)	0.727	0.727	0.726	0.726
	Internal test	0.729 (0.574–0.859)	0.574	0.573	0.563	0.568
	External test	0.653 (0.503–0.786)	0.438	0.517	0.418	0.462
PP						
	Training	0.827 (0.770–0.878)	0.637	0.645	0.633	0.639
	Internal test	0.678 (0.524–0.809)	0.475	0.477	0.464	0.470
	External test	0.586 (0.425–0.723)	0.484	0.590	0.544	0.566
Combined						
	Training	0.909 (0.866–0.946)	0.780	0.786	0.777	0.782
	Internal test	0.737 (0.588–0.857)	0.525	0.511	0.513	0.512
	External test	0.679 (0.525–0.808)	0.516	0.749	0.728	0.738
vgg19						
AP	Training	0.998 (0.994–1.000)	0.959	0.961	0.959	0.960
	Internal test	0.976 (0.936–1.000)	0.919	0.926	0.921	0.924
	External test	0.919 (0.831–0.980)	0.781	0.785	0.776	0.780
PP						
	Training	0.998 (0.995–1.000)	0.963	0.964	0.964	0.964
	Internal test	0.947 (0.874–0.994)	0.852	0.866	0.856	0.861
	External test	0.908 (0.821–0.975)	0.766	0.785	0.769	0.777
Combined						
	Training	0.999 (0.996–1.000)	0.960	0.960	0.956	0.958
	Internal test	0.982 (0.954–1.000)	0.920	0.921	0.921	0.921
	External test	0.934 (0.863–0.986)	0.828	0.831	0.827	0.829
googlenet						
AP	Training	0.970 (0.945–0.989)	0.873	0.873	0.873	0.873
	Internal test	0.912 (0.823–0.977)	0.806	0.806	0.808	0.805
	External test	0.866 (0.762–0.948)	0.751	0.758	0.740	0.749
PP						
	Training	0.983 (0.968–0.995)	0.878	0.88	0.877	0.878
	Internal test	0.888 (0.783–0.970)	0.754	0.759	0.758	0.759
	External test	0.868 (0.761–0.952)	0.719	0.721	0.705	0.713
Combined						
	Training	0.990 (0.980–0.998)	0.910	0.913	0.910	0.911
	Internal test	0.939 (0.870–0.989)	0.852	0.856	0.857	0.856
	External test	0.882 (0.781–0.965)	0.781	0.785	0.774	0.779
inception_v3						
AP	Training	0.938 (0.904–0.967)	0.816	0.821	0.816	0.818
	Internal test	0.823 (0.688–0.933)	0.705	0.735	0.710	0.722
	External test	0.741 (0.599–0.859)	0.547	0.553	0.538	0.545
PP						
	Training	0.869 (0.820–0.913)	0.714	0.719	0.716	0.717
	Internal test	0.857 (0.757–0.937)	0.620	0.654	0.629	0.641
	External test	0.802 (0.672–0.912)	0.641	0.638	0.636	0.637
Combined						
	Training	0.936 (0.903–0.965)	0.820	0.822	0.817	0.819
	Internal test	0.817 (0.692–0.919)	0.689	0.694	0.694	0.694
	External test	0.817 (0.697–0.914)	0.641	0.623	0.625	0.624

*ViT* vision transformer, *AP* arterial phase, *PP* portal venous phase, *Macro-AUC* macro-average area under the curve, *CI* confidence interval

### Fusion model construction

**Fig. 5:**
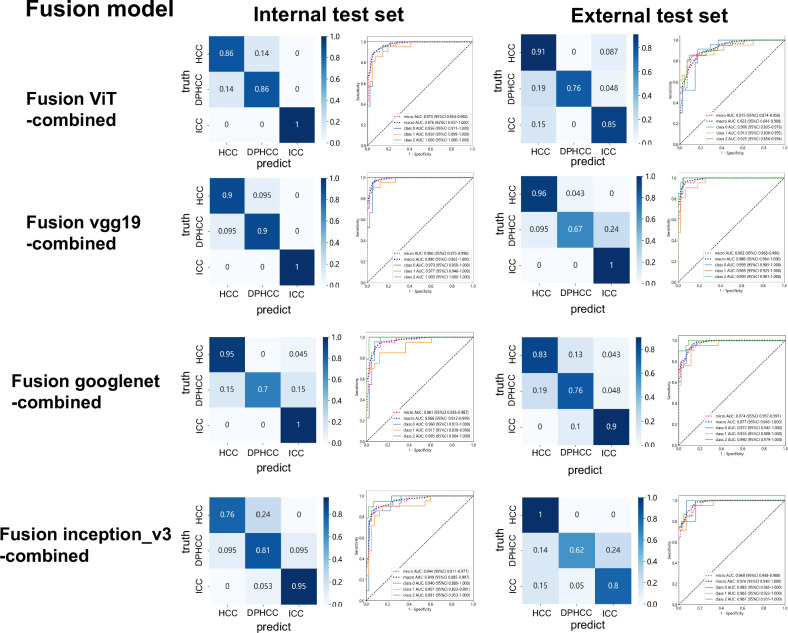
Differential diagnosis of HCC, DPHCC, and ICC based on fusion combined models. Confusion matrices and ROC curves of fusion-ViT-combined, fusion-vgg19-combined, fusion-googlenet-combined, and fusion-inception_v3-combined models in the internal and external test sets. Class 0, HCC; class 1, DPHCC; and class 2, ICC. HCC, hepatocellular carcinoma; DPHCC, dual-phenotype hepatocellular carcinoma; ICC, intrahepatic cholangiocarcinoma; AP, arterial phase; PP, portal venous phase; ROC, receiver operating characteristic; AUC, area under the curve. CI, confidence interval

In order to further improve the predictive power, we constructed the fusion models with radiomic and DTL features. After feature selection and fusion, the features with correlation coefficients > 0.9 were removed. The confusion matrices and ROC curves in the internal and external test sets of the Fusion-AP and Fusion-PP models are shown in Figs. S6 and  S7. The Fusion-vgg19-AP model showed better diagnostic accuracy. The specific details of the Fusion-AP and Fusion-PP models are shown in Table S1. Furthermore, we constructed the Fusion-combined models to improve diagnostic accuracy. The Fusion-ViT-combined and Fusion-vgg19-combined models have better diagnostic performance than other models, especially the Fusion-vgg19-combined model. The confusion matrices and ROC curves of different Fusion-combined models are shown in Fig. 5. Diagnostic accuracy rate and F1-score of the fusion-vgg19-combined model > 0.90 in the internal test set and > 0.85 in the external test set, respectively. For the Fusion-vgg19-combined model, the macro-AUC (95% CI), accuracy, and F1-score were 1.000 (1.000–1.000), 0.984, and 0.983 in the training set, 0.990 (0.965–1.000), 0.935, and 0.937 in the internal test set, and 0.988 (0.964–1.000), 0.875, and 0.885 in the external test set. The specific details of fusion-combined models are shown in Table 4.

**Table 4:** Performances of the fusion combined models

Fusion combined model		Macro-AUC (95% CI)	Accuracy	Precision	Recall	F1-score
ViT						
	Training	1.000 (0.999–1.000)	0.975	0.978	0.975	0.976
	Internal test	0.976 (0.937–1.000)	0.903	0.905	0.905	0.905
	External test	0.923 (0.841–0.988)	0.844	0.871	0.830	0.850
vgg19						
	Training	1.000 (1.000–1.000)	0.984	0.984	0.983	0.983
	Internal test	0.990 (0.965–1.000)	0.935	0.937	0.937	0.937
	External test	0.988 (0.964–1.000)	0.875	0.899	0.872	0.885
googlenet						
	Training	0.999 (0.998–1.000)	0.967	0.970	0.967	0.968
	Internal test	0.996 (0.912–0.999)	0.885	0.901	0.889	0.895
	External test	0.977 (0.943–1.000)	0.828	0.821	0.834	0.828
inception_v3						
	Training	0.995 (0.989–0.999)	0.931	0.931	0.931	0.931
	Internal test	0.949 (0.885–0.997)	0.836	0.844	0.840	0.842
	External test	0.976 (0.940–1.000)	0.812	0.845	0.800	0.822

*ViT* vision transformer, *Macro-AUC* macro-average area under the curve, *CI* confidence interval

### DTL networks visualization

**Fig. 6:**
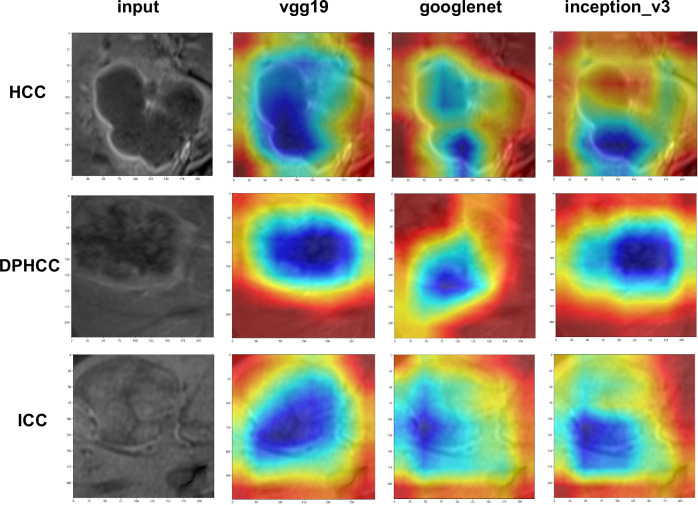
Visualization of three patient examples. HCC, DPHCC, and ICC examples show the gray-scale CE-MRI image and corresponding Grad-CAM heatmap of DTL models. Grad-CAM heatmaps show that the low echo area inside the tumor is valuable for differential diagnosis of malignant hepatic tumors. HCC, hepatocellular carcinoma; DPHCC, dual-phenotype hepatocellular carcinoma; ICC, intrahepatic cholangiocarcinoma; CE-MRI, contrast-enhanced MRI; DTL, deep transfer learning; Grad-CAM, gradient-weighted class activation mapping

The DTL networks were visualized by the Grad-CAM heatmaps. The feature heatmaps generated by the last convolutional layer in vgg19, googlenet, and inception_v3 models were visualized in Fig. 6. The Grad-CAM heatmaps for visually perceptible tumor regions capture a majority of the image details. The reliability of transfer learning in feature extraction is confirmed to some extent. Visualizing the output of the three networks helps to understand why the DTL models can correctly discriminate DPHCC from HCC and ICC.

## Discussion

In this paper, we proposed that CE-MRI-based radiomics and DTL models were developed and validated to distinguish the HCC, DPHCC, and ICC. Preoperative identification of these three malignant hepatic tumors is crucial for guiding targeted treatment and interventions. Our results consisted of 381 patients in four centers. In addition to single models, we established multi-phase fusion models to enhance diagnostic performance. Additionally, we evaluated the diagnosis precision for each HCC, DPHCC, and ICC, which can further demonstrate the stability and superiority of model prediction.

With continued changes in the trends of diagnosis and treatment of liver malignancies, the treatment options for various types of liver tumors are all different [31]. In recent years, the heterogeneity of tumors in terms of biological and genomic characteristics has become an important topic in cancer research [32–34]. DPHCC, a subtype of HCC, is morphologically similar to HCC but is able to co-express markers of HCC and ICC, reflecting the histopathologic heterogeneity of tumor [35–37]. However, clinicians are not able to differentially diagnose three types of liver malignancies based on MRI using the naked eye. Dong et al and Wiesinger et al utilized CEUS and MRI features for preoperative differential diagnosis between HCC and ICC [38, 39]. Gu et al employed laboratory and clinical indicators along with MRI imaging features to differentially diagnose between DPHCC and HCC, as well as DPHCC and ICC. However, as of now, there is a lack of an automated machine-learning model for the preoperative discrimination among these three types of liver malignancies [9]. Therefore, it is valuable to develop a non-invasive automated prediction model to differentially diagnose different liver malignancies to help clinicians make better-personalized treatment suggestions and help patients make better treatment decisions.

In recent years, with the introduction of artificial intelligence, some recent studies have shown that machine learning can be well applied to the medical field, it can assist doctors in preoperative disease prediction and identify different disease subtypes by ultrasound, CT, MRI, etc. [40, 41]. Yu et al reported that radiomics machine learning models showed high predictive accuracy and stability for predicting vessels encapsulating tumor clusters in hepatocellular carcinoma [42]. Yun Bian et al extracted MRI images and developed a deep-learning AI model to predict lymph node metastasis in pancreatic cancer [43]. It has also been shown that deep learning models provided a good predictive efficacy in identifying hepatic malignancies and are able to discriminate HCC, ICC, and metastatic hepatocellular carcinoma [44], but there is no report on the discrimination of DPHCC, HCC, and ICC.

Therefore, we established and validated radiomics and DTL models for the discrimination of DPHCC, HCC, and ICC based on CE-MRI in the arterial and portal phases. Notably, the advantages of both radiomics and deep learning technologies are not only that the data are easily accessible but also that they are non-invasive for the patients [33, 34]. In DL models, neural networks exist overfitting problems, especially when the number of samples used in the training set is small, the AUC performance on the training set is good, but the generalization ability of the test set data is poor. To solve this problem, it is important to maximize the size of training and test samples. Unlike previous deep learning models, we segmented the AP and PP CE-MRI images of the final inclusion of 381 patients from four centers, segmenting the *n* + 1, *n* + 2, *n* − 1, and *n* − 2 layers of the images in addition to segmenting the maximum ROI layer, which preserved the heterogeneity of the malignant tumors to a greater extent. Concurrently, five 2D images per one 3D CE-MRI image was acquired, and ultimately 381 patients were able to obtain 3810 2D images. DTL features are extracted from each 2D image, and then the extracted features are homogenized to build DTL models afterward. Compared to the CNN model, the essential feature of ViT is its direct global relationship modeling, which increases the sensory area of the image for more contextual information, which is why many researchers have switched from CNNs to ViT, and the self-attention of ViT can be characterized by robust global relationship modeling. Some studies showed that compared with CNN models, the ViT model has better generalization capacity and predictive performance [26, 45]. However, in our study, the vgg19 models were better than other models in most cases, including the ViT model, showing superior discriminatory ability and clinical application prospects. Especially, in the external test set, the vgg19-combined model achieved an AUC of 0.934 (95% CI: 0.863–0.986), an accuracy of 0.828, and an F1-score of 0.829, in comparison, the ViT-combined model demonstrated an AUC of 0.679 (95% CI: 0.525–0.808), the accuracy of 0.516, and an F1-score of 0.738. This may, in part, be due to the low sample size. ViT models use self-attention mechanisms to process images, which may require more data to learn effective image representations. In contrast, CNN models, with convolutional layers, excel at capturing local features, aiding in learning from small samples. The diagnosis accuracy of both the training, internal test, and external test sets of the vgg19 models exceeds 0.75. To further improve the prediction accuracy of the model, we combined the images of AP and PP phases and fused the DTL features with the radiomics features to construct a fusion model. The advantage of the fusion model is that it retains the spatial heterogeneity information of radiomics features of tumor tissues while adding the high throughput characteristics of DTL features. The fusion model fully leverages features at different hierarchies, enhancing the model’s comprehension of images. When the fusion models were compared, the accuracy, precision, recall, and F1-score of the Fusion-vgg19-combined model were > 0.85 in the training, internal test, and external test sets, which showed the best diagnostic efficiency.

There are several limitations in this study. Firstly, this is a retrospective study and requires validation by prospective studies. Secondly, the fusion models containing radiomics and DTL features showed better predictive performance, but the interpretability of these features in the biological sense was poor. In the future, we would like to further combine genetic and transcriptomic features, and combine the segmentation of patient pathology images to build a comprehensive model to better improve the stability and interpretability of the model.

## Conclusions

In this study, radiomics and DTL models were used to effectively distinguish between three different types of liver malignancies, with the vgg19 models exhibiting superior diagnostic performance in most cases. The fusion of DTL features and radiomics features further improved the stability and accuracy of model diagnosis, indicating that building multimodal fusion models for disease diagnosis and differential diagnosis formulation was a more valuable path.

## Supplementary information

ELECTRONIC SUPPLEMENTARY MATERIAL

## Data Availability

The image data from four centers are not publicly available due to the data privacy and restricted permissions of the current study. The anonymized data are available under restricted access for patient privacy, access can be obtained by sending a request to the corresponding author CHH for academic purposes.

## Abbreviations

AFP: Alpha-fetoprotein
ALT: Alanine aminotransferase
AP: Arterial phase
AST: Aspartate aminotransferase
AUC: Area under the receiver operating characteristic curve
CE-MRI: Contrast-enhanced MRI
CNN: Convolutional neural network
DPHCC: Dual-phenotype hepatocellular carcinoma
DT: Decision tree
DTL: Deep transfer learning
GGT: Gamma-glutamyl transferase
HBV: Hepatitis B virus
HCC: Hepatocellular carcinoma
ICC: Intrahepatic cholangiocarcinoma
LASSO: Least absolute shrinkage and selection operator
LR: Logistic regression
PP: Portal venous phase
RF: Random forest
ROC: Receiver operating characteristic
SVM: Support vector machine
ViT: Vision transformer
VOI: Volume of interest
