# Supplementary material for: MRI-based deep learning radiomics to differentiate dual-phenotype hepatocellular carcinoma from HCC and intrahepatic cholangiocarcinoma: a multicenter study
Source: Insights Imaging. 2025 Jan 29;16:27. doi: 10.1186/s13244-025-01904-y (PMC11780023; doi:10.1186/s13244-025-01904-y)
Supplement: Supplementary file 1 — ELECTRONIC SUPPLEMENTARY MATERIAL [file 13244_2025_1904_MOESM1_ESM.pdf]

MRI-based deep learning radiomics to differentiate dual-phenotype hepatocellular carcinoma from HCC and intrahepatic cholangiocarcinoma: a multicenter study
ELECTRONIC SUPPLEMENTARY MATERIAL

MR imaging acquisition

The specific machines included Simens Magnetom Skyra 3.0T MRI (Siemens, Germany), GE Medical Systems 3.0T MRI (GE, USA) and Philips Medical Systems 3.0T MRI (Philips Netherlands) imaging system, a 16-channel phased array body coil. The detailed scanning sequences and parameters are shown in the tables below. Diffusion-Weighted Imaging (DWI) was acquired with b values of 0 and 800 s/mm2. Gadolinium meglumine (Bayer,Berlin, Germany) was injected with a high-pressure syringe at a flow rate of 2.5-3.0 mL/s with a total dose of 0.2 mmol/kg. Arterial phase and portal venous phase images were acquired at 25 ~ 30s and 55 ~ 60s after injection of the contrast agent.

The MRI scan sequences and parameters for Siemens Medical Systems 3.0T MRI

Sequence	Category	TR(ms)	TE(ms)	FOV (mm)	Thickness (mm)	Fat saturation	Breath-hold
T1WI in/ opposed phase	3D VIBE	4.11	2.47/1.24	400×400	3.5	No	Yes
T1WI-fs	3D VIBE	4.11	1.24	400×400	3.5	Yes	Yes
T2WI-fs	TSE	3000	82	400×400	6.5	Yes	Yes
DWI	Ep2d	5600	58	400×400	6.5	Yes	No
Contrast-enhanced							
T1WI-fs （AP）	3D VIBE	4.15	2.01	400×400	3.5	Yes	Yes
T1WI-fs （PP）	3D VIBE	4.15	2.01	400×400	3.5	Yes	Yes

FOV=field of view; 3D VIBE=a three-dimensional volume interpolated breath-hold examination; Ep2d=a two-dimensional echo-planar technique; fs=fat suppression; AP=arterial phase; PP=portal venous phase

The MRI scan sequences and parameters for GE Medical Systems 3.0T MRI

Sequence	Category	TR(ms)	TE(ms)	FOV (mm)	Thickness (mm)	Fat saturation	Breath-hold
T1WI in/ opposed phase	Fast SPGR	4.6	1.2	400×360	3	No	Yes
T1WI-fs	LAVA-Flex	4.5	1.4	380×342	2.5	Yes	Yes
T2WI-fs	FRFSE-XL	7500	86.2	400×300	2	Yes	Yes
DWI	DW EPI	10000	57.1	400×320	2	Yes	No
Contrast-enhanced							
T1WI-fs (AP)	LAVA-Flex	4.6	1.7	380×342	2.5	Yes	Yes
T1WI-fs (PP)	LAVA-Flex	4.6	1.7	380×342	2.5	Yes	Yes

FOV=field of view; Fast SPGR=Fast Spoiled Gradient Recalled Echo; LAVA-Flex=Liver Acquisition with Volume Acceleration-Flexible; FRFSE-XL=Fast Recovery Fast Spin Echo-eXtended Echo Train Length; DW EPI=Diffusion-Weighted Echo Planar Imaging; fs=fat suppression; AP=arterial phase; PP=portal venous phase

The MRI scan sequences and parameters for Philips Medical Systems 3.0T MRI

Sequence	Category	TR(ms)	TE(ms)	FOV (mm)	Thickness (mm)	Fat saturation	Breath-hold
T1WI in/ opposed phase	mDIXON	3.7	1.32	400×300	6	No	Yes
T1WI-fs	mDIXON	3.7	1.32	400×300	6	Yes	Yes
T2WI-fs	TSE	448	70	320×364	4		
DWI	DW EPI	1783	55	300×360	5	Yes	No
Contrast-enhanced							
T1WI-fs(AP)	mDIXON	3.8	1.4	400×300	3	Yes	Yes
T1WI-fs(AP)	mDIXON	3.8	1.4	400×300	3	Yes	Yes

FOV=field of view; mDIXON=modified Dixon; fs=fat suppression; TSE=turbo spin-echo; DW EPI=Diffusion-Weighted Echo Planar Imaging; AP=arterial phase; PP=portal venous phase.

Fig. S1. The features selection and models construction based on radiomics.

Fig. S1

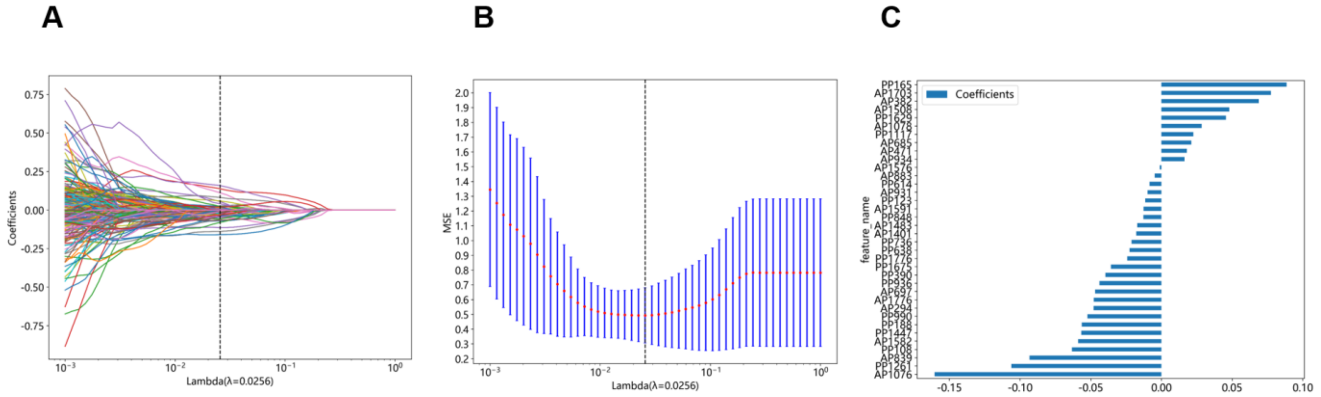

(A-C) LASSO regression for screening of radiomics features.

LASSO=the least absolute shrinkage and selection operator; AP=arterial phase; PP=portal venous phase; HCC=hepatocellular carcinoma; DPHCC=dual-phenotype hepatocellular carcinoma; ICC=intrahepatic cholangiocarcinoma.

Fig. S2. The confusion matrices for DTL models in the internal test set.

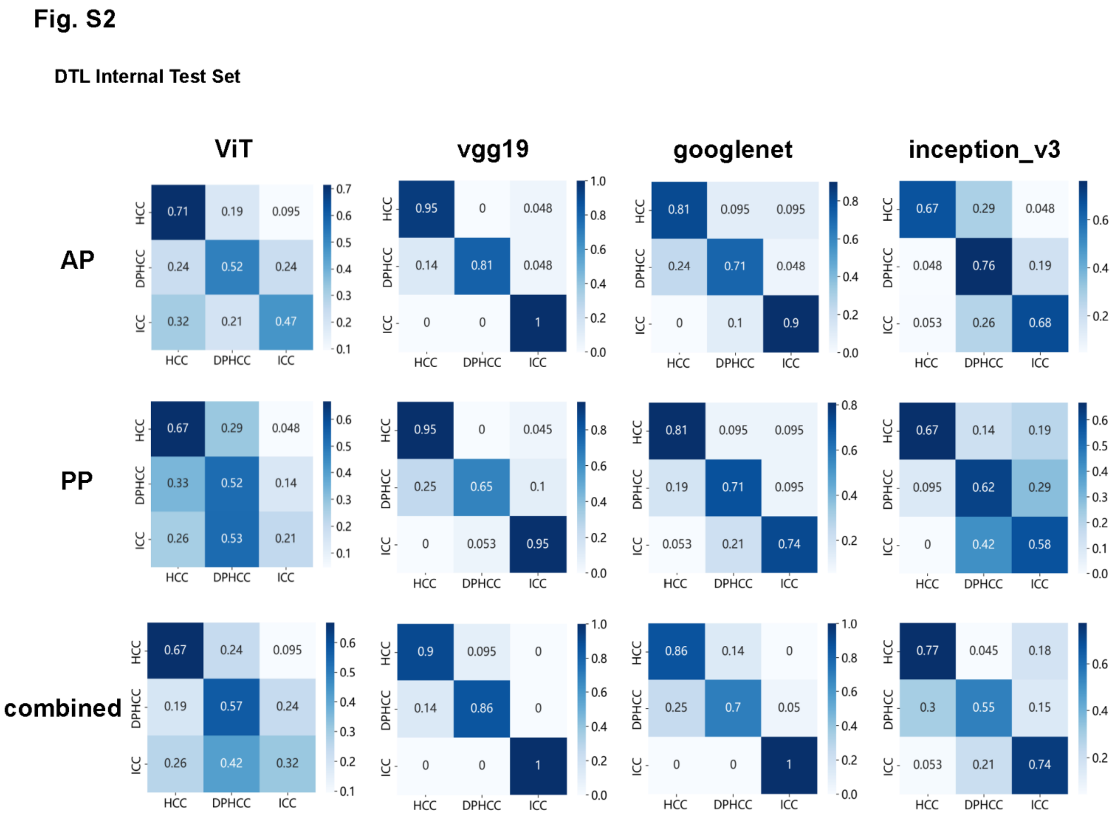

DTL=deep transfer learning; ViT=Vision Transformer; AP= arterial phase; PP=portal venous phase; HCC=hepatocellular carcinoma; DPHCC=dual-phenotype hepatocellular carcinoma; ICC=intrahepatic cholangiocarcinoma.

Fig. S3. The ROC curves for DTL models in the internal test set.

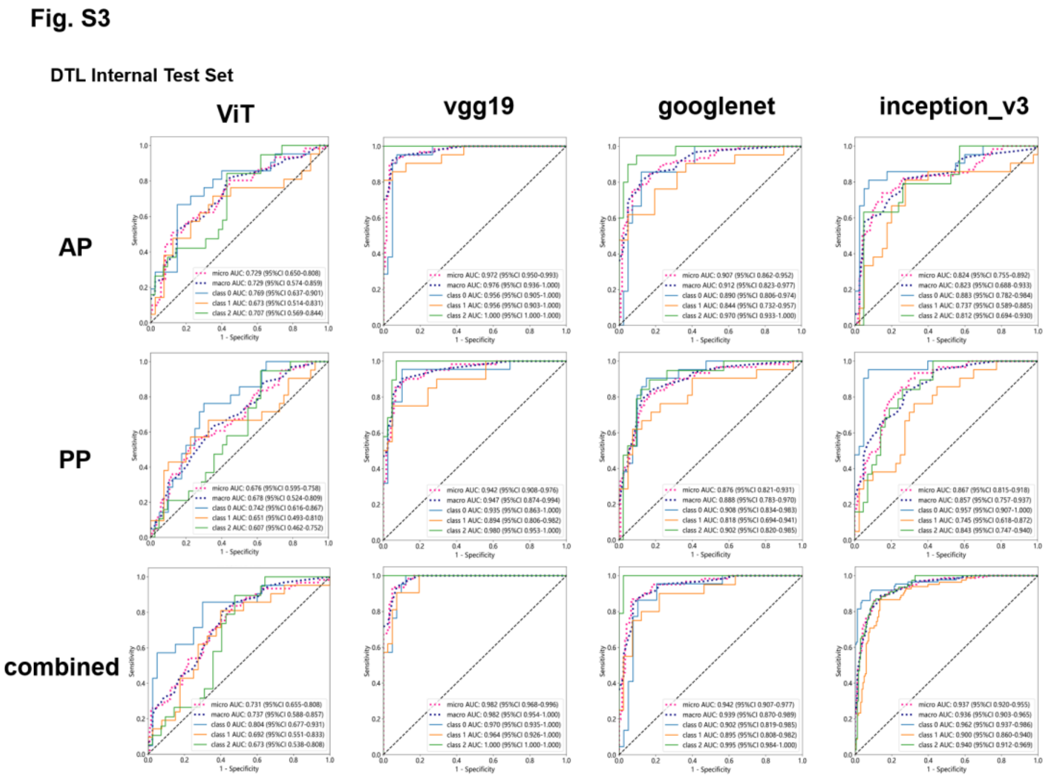

Class 0=HCC; class 1=DPHCC; class 2=ICC; DTL=deep transfer learning; ViT=Vision Transformer; AP= arterial phase; PP=portal venous phase; HCC=hepatocellular carcinoma; DPHCC=dual-phenotype hepatocellular carcinoma; ICC=intrahepatic cholangiocarcinoma; CI=confidence interval.

Fig. S4. The confusion matrices for DTL models in the external test set.

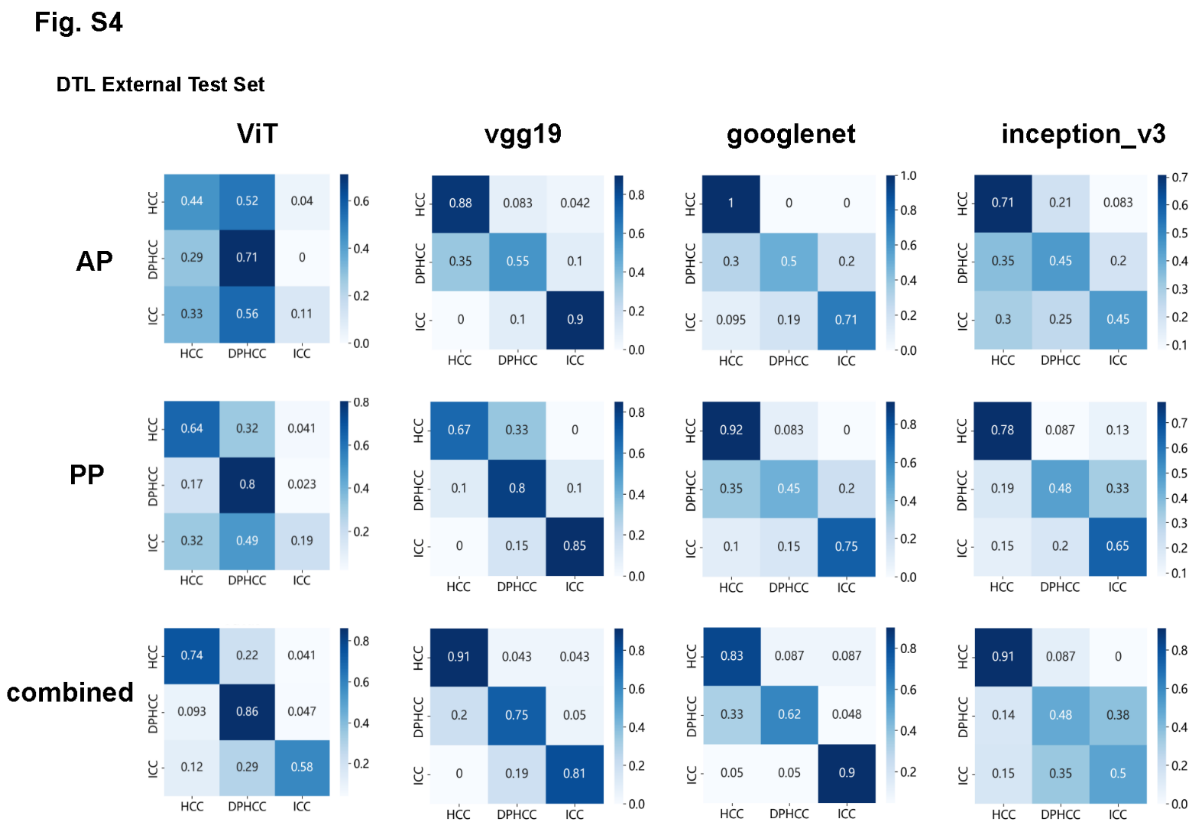

DTL=deep transfer learning; ViT=Vision Transformer; AP=arterial phase; PP=portal venous phase; HCC=hepatocellular carcinoma; DPHCC=dual-phenotype hepatocellular carcinoma; ICC=intrahepatic cholangiocarcinoma.

Fig. S5. The ROC curves for DTL models in the external test set.

Fig. S5

DTL External Test Set

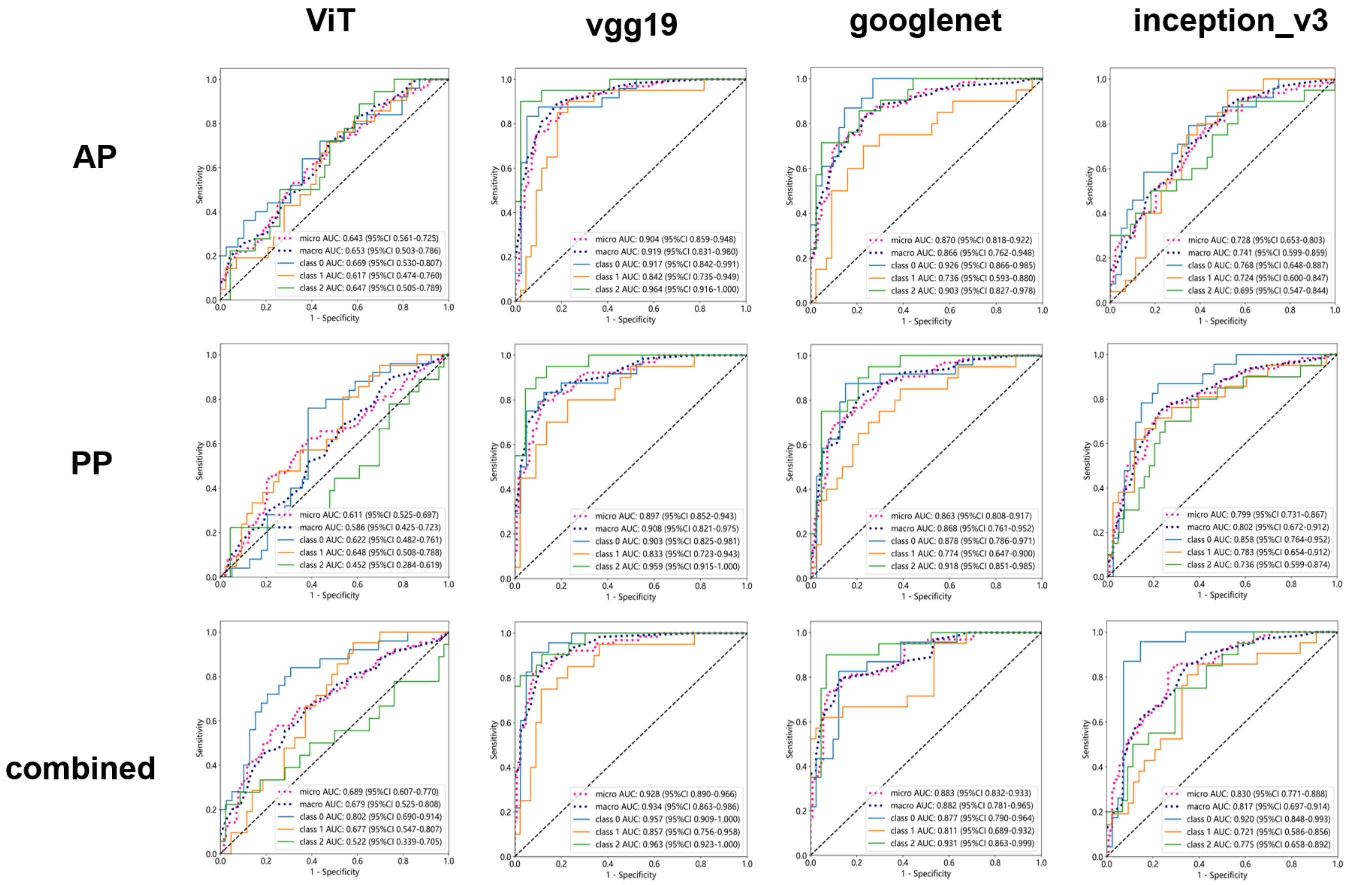

Class 0=HCC; class 1=DPHCC; class 2=ICC; DTL=deep transfer learning; ViT=Vision Transformer; AP= arterial phase; PP=portal venous phase; HCC=hepatocellular carcinoma; DPHCC=dual-phenotype hepatocellular carcinoma; ICC=intrahepatic cholangiocarcinoma; CI=confidence interval.

Fig. S6. The confusion matrices and ROC curves for fusion AP and PP models in the internal test set.

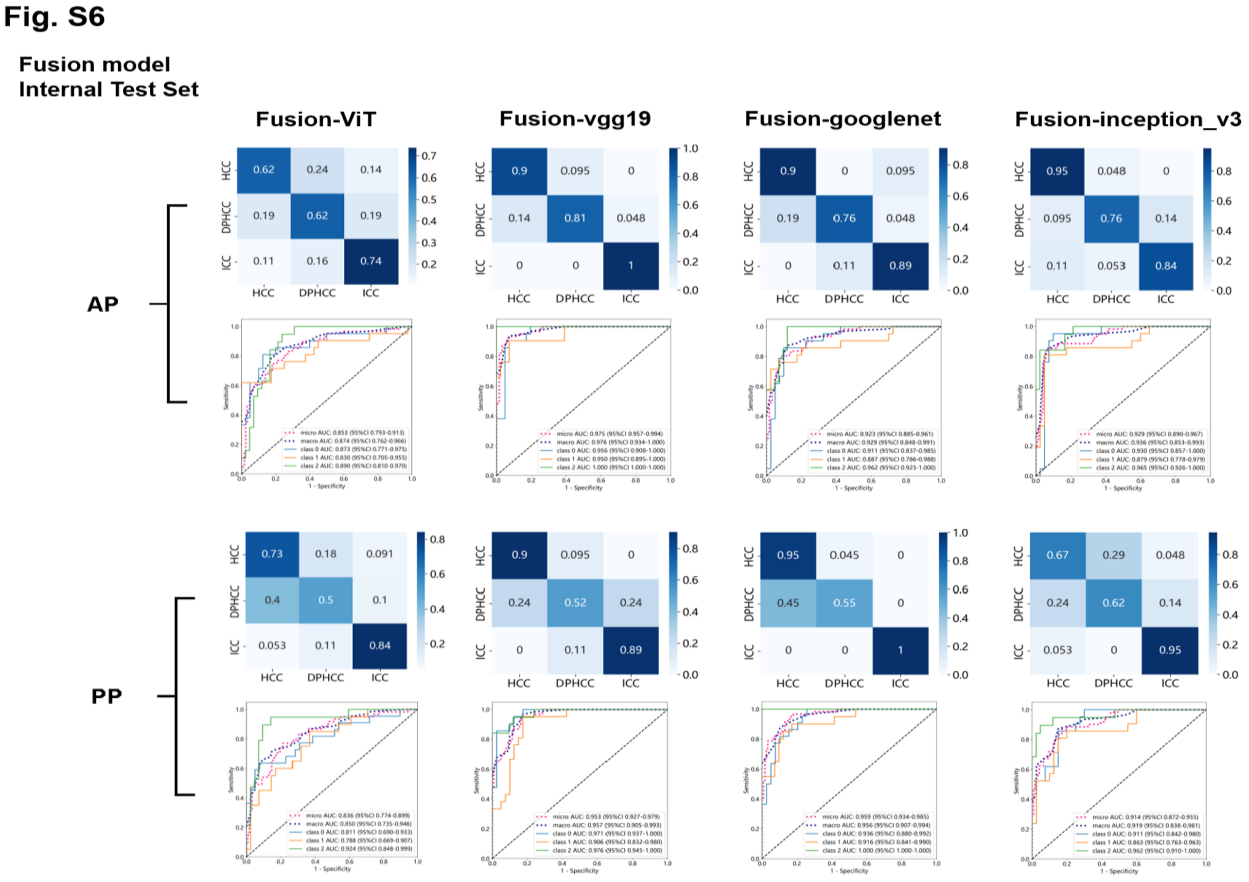

Class 0=HCC; class 1=DPHCC; class 2=ICC; DTL=deep transfer learning; ViT=Vision Transformer; AP= arterial phase; PP=portal venous phase; HCC=hepatocellular carcinoma; DPHCC=dual-phenotype hepatocellular carcinoma; ICC=intrahepatic cholangiocarcinoma; CI=confidence interval.

.

Fig. S7. The confusion matrices and ROC curves for fusion AP and PP models in the external test set.

Fig. S7

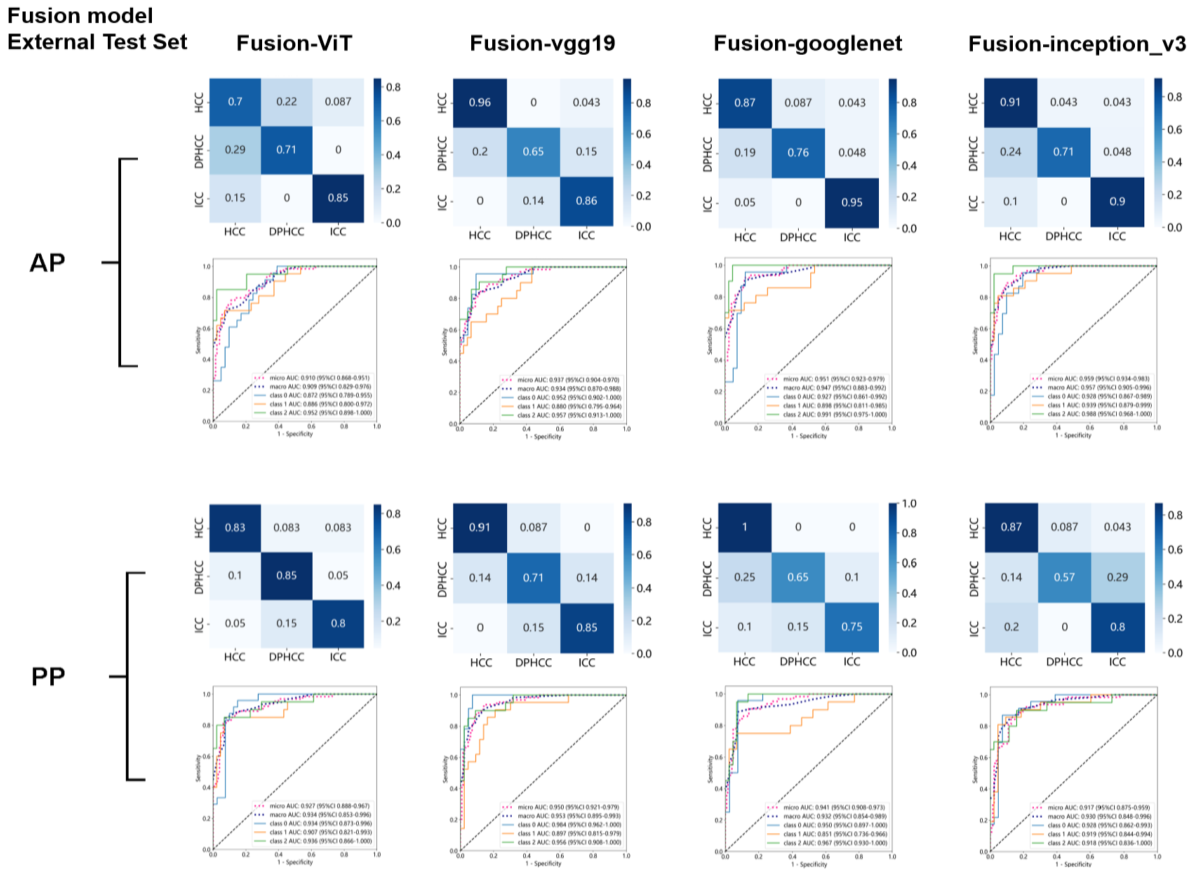

Class 0=HCC; class 1=DPHCC; class 2=ICC; DTL=deep transfer learning; ViT=Vision Transformer; AP= arterial phase; PP=portal venous phase; HCC=hepatocellular carcinoma; DPHCC=dual-phenotype hepatocellular carcinoma; ICC=intrahepatic cholangiocarcinoma; CI=confidence interval.

Table S1. Performances of the Fusion AP and PP models

Fusion model		Macro-AUC (95%CI)	Accuracy	Precision	Recall	F1-score
ViT						
AP	Training	0.977 (0.954-0.994)	0.920	0.912	0.911	0.912
	Internal test	0.874 (0.762-0.966)	0.656	0.662	0.663	0.662
	External test	0.909 (0.829-0.976)	0.750	0.668	0.739	0.702
PP						
	Training	0.968 (0.945-0.987)	0.865	0.865	0.865	0.865
	Internal test	0.850 (0.735-0.946)	0.689	0.694	0.696	0.695
	External test	0.934 (0.853-0.996)	0.828	0.834	0.837	0.836
vgg19						
AP	Training	0.999 (0.998-1.000)	0.963	0.970	0.964	0.967
	Internal test	0.976 (0.934-1.000)	0.903	0.917	0.905	0.911
	External test	0.934 (0.870-0.988)	0.828	0.830	0.821	0.826
PP						
	Training	1.000 (1.000-1.000)	0.992	0.991	0.991	0.991
	Internal test	0.957 (0.905-0.993)	0.770	0.769	0.943	0.847
	External test	0.953 (0.895-0.993)	0.828	0.814	0.813	0.814
googlenet						
AP	Training	0.995 (0.989-0.999)	0.922	0.927	0.923	0.925
	Internal test	0.929 (0.848-0.991)	0.852	0.857	0.856	0.856
	External test	0.947 (0.883-0.992)	0.859	0.830	0.859	0.844
PP						
	Training	0.996 (0.992-1.000)	0.963	0.964	0.963	0.963
	Internal test	0.956 (0.907-0.994)	0.836	0.871	0.841	0.856
	External test	0.932 (0.854-0.989)	0.812	0.820	0.794	0.806

inception_v3						
AP	Training	0.990 (0.978-0.999)	0.902	0.904	0.902	0.903
	Internal test	0.936 (0.853-0.993)	0.852	0.857	0.855	0.856
	External test	0.957 (0.905-0.996)	0.844	0.875	0.839	0.856
PP						
	Training	0.967 (0.943-0.987)	0.882	0.882	0.881	0.881
	Internal test	0.919 (0.838-0.981)	0.738	0.737	0.745	0.741
	External test	0.930 (0.848-0.996)	0.750	0.764	0.739	0.751

ViT=Vision Transformer; AP=arterial phase; PP=portal venous phase; Macro-AUC=macro-average area under the curve; CI=confidence interval.
